# Thrombus Degradation by Fibrinolytic Enzyme of* Stenotrophomonas* sp. Originated from Indonesian Soybean-Based Fermented Food on Wistar Rats

**DOI:** 10.1155/2016/4206908

**Published:** 2016-08-21

**Authors:** Florensia Nailufar, Raymond R. Tjandrawinata, Maggy T. Suhartono

**Affiliations:** ^1^Dexa Laboratories of Biomolecular Sciences, Jalan Industri Selatan V Blok PP/7, Jababeka II, Cikarang 17550, Indonesia; ^2^Faculty of Biotechnology, Atmajaya University, Jalan Jenderal Sudirman 51, Jakarta Selatan 12930, Indonesia; ^3^Department of Food Science and Technology, Faculty of Agricultural Engineering and Technology, Bogor Agricultural University, Bogor 16002, Indonesia

## Abstract

*Objective. *To evaluate thrombus degrading effect of a fibrinolytic enzyme from food origin* Stenotrophomonas *sp. of Indonesia.* Methods*. Prior to animal study, the enzyme safety was tested using cell culture. The effect on expression of tissue plasminogen activator was also analysed in the cell culture. For* in vivo *studies, 25 Wistar rats were used: normal control, negative control, treatment groups with crude and semipurified enzyme given orally at 25 mg/kg, and positive control group which received Lumbrokinase at 25 mg/kg. Blood clot in the tail was induced by kappa carrageenan injection at 1 mg/kg BW.* Results*. Experiment with cell culture confirmed the enzyme safety at the concentration used and increased expression of tPA. Decreasing of thrombus was observed in the positive group down to 70.35 ± 23.11% of the negative control animals (100%). The thrombus observed in the crude enzyme treatment was down to 56.99 ± 15.95% and 71.5 ± 15.7% for semipurified enzyme. Scanning electron microscopy showed clearly that bood clots were found in the animals injected with kappa carrageenan; however, in the treatment and positive groups, the clot was much reduced.* Conclusions*. Oral treatment of enzyme from* Stenotrophomonas *sp. of Indonesian fermented food was capable of degrading thrombus induced in Wistar rats.

## 1. Introduction

Cardiovascular diseases are reported as the leading cause of 17.5 million people deaths [1]. By 2030, more than 23 million people estimated will die each year due to cardiovascular disease, with acute myocardium infarction because of thrombosis of blood vessels [2]. The present drug, human tissue plasminogen activator (t-PA), is a major activator of the extrinsic fibrinolytic system [3] and a member of serine protease family, which is involved in fibrinolysis [4]. Thrombolytic drug which dissolve fibrin in the blood clots are used not only for myocardial infarction but also for thromboembolic strokes, deep vein thrombosis, and pulmonary embolism to clear the blockage of blood vessel [5, 6]. Thrombolytic therapy with t-PA at present is limited by the relatively high incidence of reocclusion and resistance to reperfusion, despite therapeutic heparinization [7, 8].

Tissue plasminogen activators, streptokinase and urokinase, activate plasminogen into active plasmin, which further degrade fibrin in the blood clots. Another potent thrombolytic agent lumbrokinase of the earth worm* Lumbricus* and lumbrokinase-like proteins degrade fibrin directly [8–10].

Streptokinase and urokinase are also considered effective drugs for thrombolytic therapy. However, beside being expensive, the side effects of the drugs such as occurrence of allergic reactions due to streptokinase administration have been reported. Attempts to finding new and safe drugs of more natural origin are thus being actively pursued.

Microorganisms have been recognized as source of thrombolytic agent, such as streptokinase from* Streptococcus hemolyticus *and staphylokinase from* Staphylococcus aureus *[2]. More attention is given to microbial fibrinolytic enzymes of food origin, in particular, the traditional fermented foods, which have been consumed safely for decades. Later discovery reported potent thrombolytic agent nattokinase (NK) from* Bacillus natto* isolated from Japanese fermented soybean food [2, 11]. In addition,* Bacillus amyloliquefaciens* DC-4 and* Bacillus subtilis* LD-8547 isolated from Chinese soybean-fermented food called* Douchi* were also found to produce thrombolytic enzymes [12, 13].

Screening and isolation of fibrinolytic bacteria from* Oncom*, an Indonesian soybean-based fermented food, revealed several bacteria, and among these bacteria we found* Stenotrophomonas* sp. This finding is unique, because most of fibrinolytic microorganism of food origin reported belongs to* Bacillus* sp. Safety of the bacterial enzyme from the Indonesian fermented* Oncom* was tested using cell culture and experimental rats, while the effect of* Stenotrophomonas* enzyme on degrading thrombus was observed using the experimental rats.

## 2. Materials and Methods

Three bacterial isolates from* Oncom* were obtained from Bogor Agricultural University, Indonesia. *Κ*appa carrageenan and fetal calf serum (FCS) were purchased from Sigma-Aldrich, human cervix adenocarcinoma cell line (HeLa S3) and fibroblast* Mus musculus *cell line (3T3-SA) were purchased from the American Type Culture Collection (Rockville, MD, USA), and Dulbecco's Modified Eagle's Medium/F12 basal medium (Gibco, Carlsbad, CA, USA) and penicillin-streptomycin (Gibco) were obtained from their local representatives. Platelet count, erythrocyte, white blood cell, platelet distribution width, and mean corpuscular volume were determined by semiautomated Hematology Analyzer MEK-6450K (Nihon Kohden, Japan). Cell pack diluents (Nihon Kohden, Japan), autofine coater JEOL JFC 1600, and scanning electron microscopy JEOL JSM-6510 were provided by Dexa Laboratories of Biomolecular Sciences, Dexa Medica.

### 2.1. Production of Enzyme

The bacterial isolates were grown in casein medium, consisting of 0.5% (w/v) casein, 0.5% (w/v) glucose, 0.6% (w/v) Na_2_HPO_4_ 2H_2_O, 0.2% (w/v) yeast extract, 0.1% (w/v) KCl, and 0.01% (w/v) MgSO_4_ 7H_2_O; pH of the media was adjusted to 8.5. Incubation was performed overnight at 37°C and 120 rpm. Absorbance was measured at 600 nm to follow the bacterial growth. Extracellular enzyme was harvested following centrifugation at 8930 g for 30 minutes. The pellet was removed, and the supernatant (crude enzyme) was kept at −20°C until use. Ammonium sulphate (65% w/v) was added into the crude enzyme and kept at 4°C, stirred overnight, and centrifuged at 15880 g for 15 minutes at 4°C. Supernatant was removed and the pellet was collected and dissolved with phosphate buffer 20 mM, pH 7.5. The enzyme activity was analyzed following fibrin degradation assay [14], while the protein concentration was assayed following Lowry method [15].

### 2.2. Cell Cultures and Treatment

HeLa S3 cells were cultured in F12 basal medium, while 3T3 SA cells were cultured in Dulbecco's Modified Eagle's Medium supplemented with 10% fetal calf serum and 1% penicillin-streptomycin. The cells were incubated at 37°C in 5% CO_2_ atmosphere. The medium was replaced every 2-3 days until the cells reached 80% confluence. The culture was then subcultivated at a ratio of 1 : 4 using 1 mL trypsin-ethylenediaminetetraacetic acid (Gibco). Before treatment, the cells were maintained in serum-free medium. Treatment with crude and semipurified enzyme was given at various concentrations. The experiment was repeated four times for each concentration tested, and duration of treatment was 24 hours.

### 2.3. MTT (Microculture Tetrazolium Salt) Assay

FCS, L-glutamine, sodium bicarbonate, penicillin, and streptomycin were added to Dulbecco's Modified Eagle's/F12 basal medium. HeLa S3 and 3T3 SA cells were multiplied and subcultured in 75 cm^2^ culture flask (Falcon, BD) in these media, at 37°C under partial pressure of 5% CO_2_. The multiplied HeLa S3 and 3T3 SA cells were separated by using trypsin-ethylenediaminetetraacetic acid and suspended in fresh media. After reaching 80% confluent state, the cell was serum-starved before treatment with crude and semipurified enzyme. The cells were incubated with various concentrations (0, 1, 3, 5, 10, 20, 40, 80, 160, 320, and 640 *μ*g/mL) of enzymes from* Stenotrophomonas* sp.,* Bacillus cereus,* and* Bacillus licheniformis *for 24 hours. At the end of incubation, 20 *μ*L of MTT was added, and incubation was continued for another 4 hours. Finally, the plate was read using a microplate reader (BIO-RAD, USA) at 590 nm.

### 2.4. Reverse Transcription Polymerase Chain Reaction

HeLa S3 cells were cultured with FCS 10% until reaching the confluent state, treated with crude and semipurified enzyme for 24 hours, and further incubated in 5% CO_2_ at 37°C. Total RNA was extracted from HeLa S3 cells using TRIzol reagent (Invitrogen, Carlsbad, CA, USA) according to the manufacturer's recommendation. The RNA concentration was quantified using a NanoDrop 2000c spectrophotometer (Thermo Fisher Scientific, Waltham, MA, USA). Sequences of the primers used for PCR analysis of tPA gene were as follows: for forward and reverse primers: 5′-ATC TTG GGC AGA ACA TAC CG-3′ and 5′-TGC ACT CTT CCC TCT CCT GT-3′, respectively. For internal standard using beta actin gene, they were 5′-GAG TCA ACG GAT TTG GTC G-3′ and 5′-TCG CTG TTG AAG TCA GAG GA-3′ as forward and reverse primers [16]. Total PCR volume was 25 *μ*L containing 5 *μ*L DNA, Master Mix (Promega) 12.5 *μ*L, and nuclease free water 1.9 *μ*L. The cycle was as described by Medcalf et al., 1990 [17]. 5 *μ*L of PCR products was analyzed by electrophoresis with 1% agarose in TAE 1x buffer and run for 60 minutes 80 V. Quantitative RT-PCR was performed using a ChemiDoc*™* (Bio-Rad).

### 2.5. Animals

Twenty-five male rats (*Rattus norvegicus*), stock Wistar, weighing 300–400 grams were acclimatized for 7 days in an environmentally controlled room, under protocol number DIS-DLBS-PROC-APC-035. The protocol has been reviewed and approved by the Institutional Animal Care and Use Committee of Dexa Laboratories of Biomolecular Sciences.

All procedures complied with Standard Operating Procedures and Working Instruction in Animal Pharmacology Laboratories, in accordance with the Guide for the Care and Use of Laboratory Animals [18]; the facilities and programs used have been accredited by AAALAC International. Rats with tail longer than 13 cm were selected. The tail was ligated with silk 4/0, and the animals were injected with kappa carrageenan, body weight of 1 mg/kg. After 10 minutes, the ligature was removed, and the animals were observed for another 24 hours. The rats were divided into 5 (five) groups: (1) normal control group: injected (i.v.) with aqua as placebo and treated orally with normal saline three times a day, for 8 days; (2) negative control group: injected (i.v.) with kappa carrageenan, body weight of 1 mg/kg, and treated orally with normal saline three times a day for 8 days; (3) positive control group: injected (i.v.) with kappa carrageenan, body weight of 1 mg/kg (i.v.), and treated orally with lumbrokinase at a dose of 25 mg/kg body weight, three times a day for 8 days; (4) treatment group with crude enzyme: injected (i.v.) with kappa carrageenan, 1 mg/kg body weight, and treated orally with crude enzyme at a dose of 25 mg/kg body weight, three times a day for 8 days; (5) treatment group with semipurified enzyme: injected with kappa carrageenan, body weight of 1 mg/kg, and treated orally with semipurified enzyme at a dose of 25 mg/kg body weight, three times a day for 8 days. We observed length of the infarcted tail appeared as dark color daily. Quantification of the thrombus follows the following calculation:

(1) $$\begin{array}{c} \%\;\;of\;\;thrombus\;\;area=\frac{Length\;\;of\;\;dark\;\;tail\;\;on\;\;day\;\;1-length\;\;of\;\;dark\;\;tail\;\;on\;\;day\;\;of\;\;observation}{length\;\;of\;\;dark\;\;tail\;\;on\;\;day\;\;1}. \end{array}$$

At the end of the study period, the rats were euthanized with sodium pentobarbital, body weight of 150 mg/kg (i.c.), under anesthesia (ketamine at a dose of 80 mg/kg and xylazine at a dose of 7.5 mg/kg body weight i.p.).

### 2.6. Tail Segment Preparation and Scanning Electron Microscopy

After euthanizing, tails from the experimental rats were removed from the body. The tails were sliced at a width of 0.25 cm and immersed in fixative solution. The tail segments were rinsed for 10 minutes for sequential dehydration in 50, 75, 85, and 95% and absolute ethanol. The samples were rinsed twice in acetone for 10 minutes. Dried samples were platinum coated with autofine coater JEOL JFC 1600 and examined at 5 KV in JEOL JSM-6510.

### 2.7. Statistical Analysis

Data were expressed as mean ± SEM. Statistical analysis of comparison between normal cells and cells treated with crude and semipurified enzyme was conducted using Student's* t*-test. All animal experimental results were statistically analyzed by one-way ANOVA following* post hoc* test for multiple comparisons (Tukey's or Games-Howell test) using SPSS® version 23 Statistics software. All statistical tests were at 5% significance level.

## 3. Result

Previously, the fibrinolytic activities of extracellular enzyme from microorganisms from Indonesian traditional fermented food (*Oncom*) were screened using fibrin plate and zymogram method [19] and revealed three most potential isolates. These isolates were identified using 16sRNA sequence gene analysis and revealed as* Bacillus licheniformis*,* Bacillus cereus,* and* Stenotrophomonas *sp.

**Figure 1:**
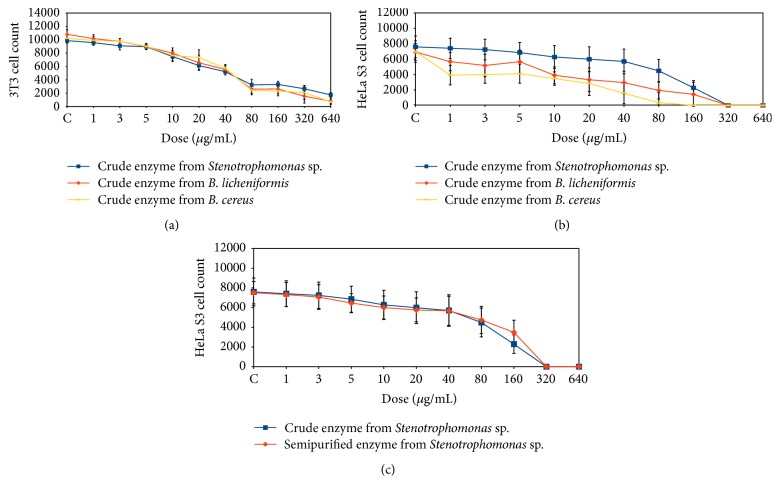
Cells count after treatments with enzyme from the three isolates. All experiments used MTT assay as mentioned in the method. 3T3 cell counts after treatment with crude enzyme (a); HeLa S3 cell counts after treatment with crude enzyme; (b) HeLa S3 cell counts after treatment with crude and semipurified enzyme from* Stenotrophomonas* sp. (c).

Enzyme from* Stenotrophomonas *sp. was selected further for PCR analysis and animal experiments due to being the least toxic (shown by MTT assay in Figure 1) and in consideration also of the previous result of the fibrin plate and zymogram analysis. Activities of the crude and ammonium sulphate precipitated (semipurified) enzymes were measured using fibrin degradation assay [14] and the protein concentrations were determined by Lowry's method [15] as shown in Table 1.

### 3.1. MTT Assay

The MTT analysis was performed as early screening for toxicity, before conducting PCR analysis and experiment on animal models. This assay is useful to test the cell viability and possible cytotoxicity of the enzymes from the three potential fibrinolytic bacteria.

MTT analysis shows different result on 3T3 and HeLa S3 cells counts following treatment of crude enzyme from isolate* Stenotrophomonas *sp.,* B. licheniformis, *and* B. cereus* (Figure 1). The 3T3 cell counts show no difference when treated with similar doses of crude enzyme from the three isolates (Figure 1(a)). The results on HeLa S3 cells were however different; the percentages of cell death after treatment with crude enzyme from* Stenotrophomonas *sp.,* B. licheniformis, *and* B. cereus *at 40 *μ*g/mL were 24.85%, 57.22% and 77.39%. These percentages were calculated as the initial cell number minus the number of remaining cells (Figure 1(b)) divided by the initial cell number times 100%. Higher percentage means higher cell death. Based on this data, further study was conducted with crude and semipurified enzyme from* Stenotrophomonas *sp. which appeared as less toxic. Similar effects on HeLa S3 cell count were found when the cells received crude and semipurified enzyme from* Stenotrophomonas *sp. (Figure 1(c)).

The MTT assay resulted in two important findings: (1) among the three potential fibrinolytic bacteria, enzyme from* Stenotrophomonas* was found as least toxic and would be used in further experiment; (2) crude and semipurified enzyme from* Stenotrophomonas *gave similar result and would be also used in further experiments with PCR to find their effect on t-PA expression and used in experimental rats to find their effect on thrombus degradation. The concentrations/doses applied in further experiments took into consideration the MTT data and previous study on fibrin plate and zymogram assay.

### 3.2. Reverse Transcription Polymerase Chain Reaction Analysis

**Figure 2:**
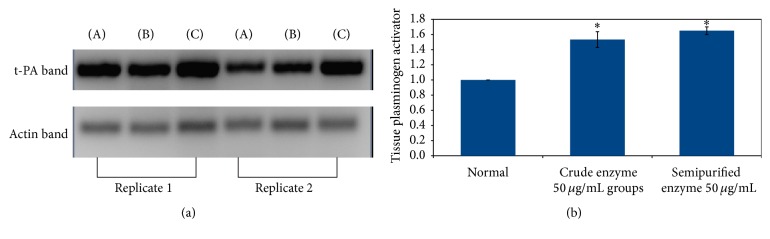
Tissue plasminogen activator expression and quantitative PCR result in HeLa S3 cells. t-PA expression gene in HeLa S3 cells (a); (A) normal control, (B) crude enzyme of* Stenotrophomonas* sp. 50 *μ*g/mL, and (C) semipurified enzyme of* Stenotrophomonas* sp. 50 *μ*g/mL; quantitative PCR (b). ^*∗*^significant difference compared with normal (*p* < 0.05).

PCR method was performed to analyze expression of tissue plasminogen activator (t-PA) gene in HeLa S3 cells following enzyme treatment. The data shows that treatment with crude and semipurified enzyme from* Stenotrophomonas* sp. increased t-PA expression compared with normal control as shown in Figure 2.

The increased expression of tissue plasminogen activator implies increase in fibrinolysis activity, due to activation of plasminogen into active plasmin.

### 3.3. Thrombolytic Activity in Experimental Animals

The efficacy of enzyme from* Stenotrophomonas* sp. to reduce thrombus formation was analyzed using experimental rats. In this study, we applied kappa carrageenan to induce thrombus formation in the rats, because, among different carrageenans, kappa carrageenan was reported as thrombogenic, whereas lambda-carrageenan were inactive in this respect [20]. As the consequence of thrombosis, tail infarction became visible some minutes after intravein administration [21].

**Figure 3:**
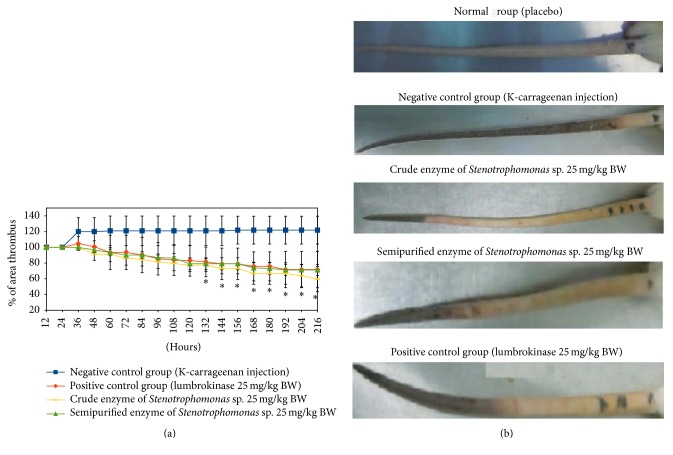
Thrombus degradation in rats tail following oral enzyme treatment. Percentage of thrombus left (a). Thrombus area on day fifth (b). ^*∗*^significance difference compared with negative control group (*p* < 0.05).

Figure 3 shows that injection of kappa carrageenan immediately induced formation of thrombus in the rats tail which appears as dark color. We measured the length of this dark segment in the tail of all experimental groups everyday. Different change of the tail infarction across treatments is shown in Figure 3(a). The percentage of tail infarction was calculated as mentioned in the method. In the negative control group, the percentage started from 100% after 12 hrs of injection and increased up to 120% on day 9. Treatment with crude enzyme, semipurified enzyme (ammonium sulphate precipitated), and lumbrokinase reduced the length of the thrombus formed, which is shown as decrease in percentage of the tail infarction. We found that, in the lumbrokinase group, the dark tail was 70.35 ± 23.11% while in the crude enzyme treatment, it reached 56.99 ± 15.95% and 71.5 ± 15.7% for the treatment with semipurified enzyme. Our observation indicated that the length of the dark tail (implying thrombus) reached 8.9 ± 3.4 cm, 8.4 ± 4.2 cm, and 10.9 ± 2.2 cm after oral treatments with lumbrokinase, crude enzyme (*Stenotrophomonas* sp.), and semipurified enzyme, respectively, while the dark tail segment in the negative control remained 13 cm.

Thrombus formed in the rat tail induced by carrageenan injection was much reduced by oral treatments of crude enzyme, semipurified enzyme of* Stenotrophomonas *sp., and also lumbrokinase (in the positive control group). At the end of the treatments period, there was sign of gangrene in the rat tail, especially in the negative control. Gangrene is a term used to describe decay or death of an organ or tissue, due to lack of blood supply. This condition was influenced by the presence of clot formation due to kappa carrageenan injection.

**Figure 4:**
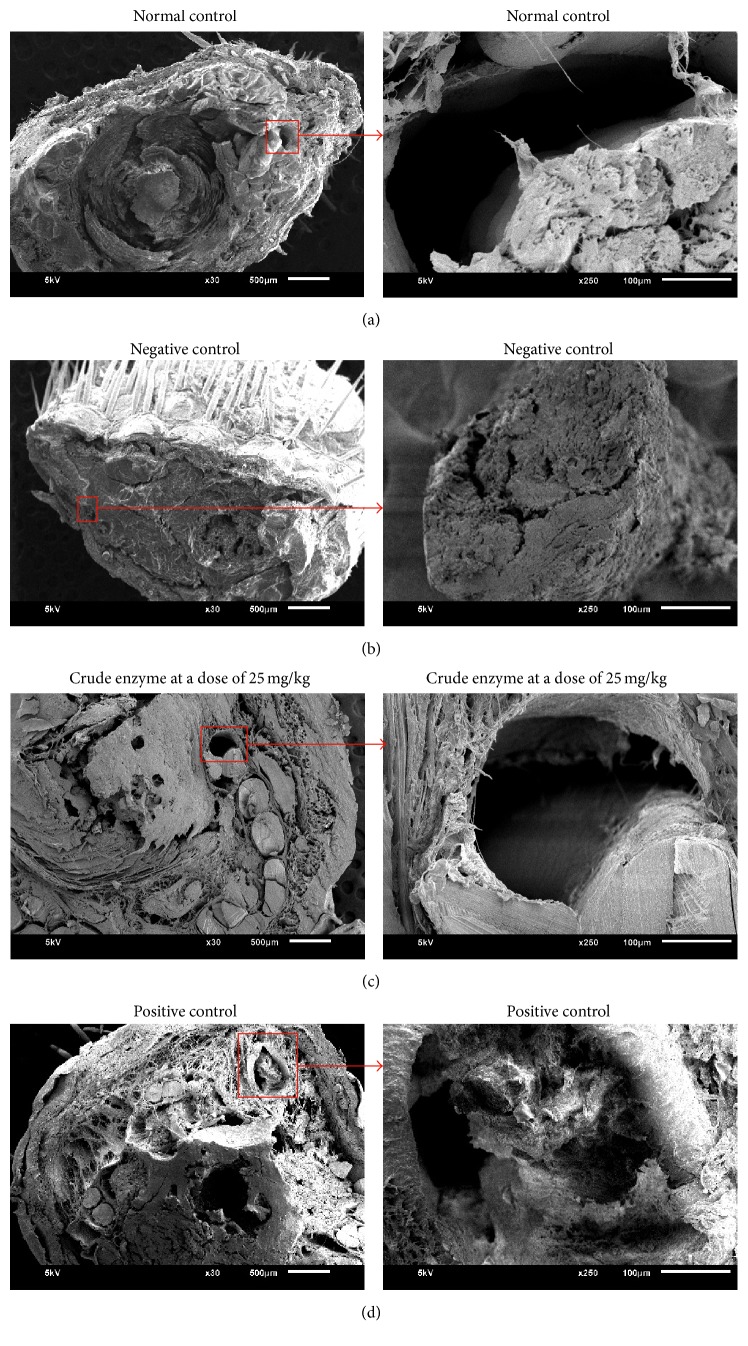
Scanning electron microscopy of the tail cross section from the experimental rats (including blood vessels). Normal control (a); negative control (kappa carrageenan injection) (b); crude enzyme of* Stenotrophomonas *sp. 25 mg/kg BW (c); positive control (lumbrokinase 25 mg/kg BW) (d).

Analysis using scanning electron microscopy was conducted at 250x magnification and focused on the tail vein, in order to visualize formation of the thrombus.  Figure 4 shows experimental results for four different treatments. The result confirms that *Κ*-carrageenan injection produced blood clots or thrombus (Figure 4(b)) while the normal control group did not reveal any blockages in the blood vessels (Figure 4(a)). There were significant differences found in the negative control and the treatment groups. The veins in negative control group were covered by thrombus, while, for treatment groups, a significant part was becoming clear indicating less or no blockage (Figures 4(c) and 4(d)).

### 3.4. Hematology

To measure the health status of the experimental rats following enzyme treatment, we performed blood analysis at the end of the experiment. The hematology data were within the normal values for* Rattus norvegicus* [22], which implies that treatments with crude and semipurified enzyme of* Stenotrophomonas *sp. did not alter the health status (Table 2). Kappa carrageenan injection influenced the platelet count statistically. Platelet or thrombocytes are involved in hemostasis, leading to formation of blood clots which was already observed in Figure 3 (dark color tail infarction). Compared with the rats in the negative control, we observed slight decrease in platelet count in all treatment groups. Within the group of semipurified enzyme, the platelet values were lower as expected and not significantly different from those of the normal group (*p* > 0.05).

## 4. Discussion

Thrombotic complications of cardiovascular disease are the main causes of death and disability in many patients. Thrombolysis have been used to lower the burden of such life-threatening diseases as myocardial infarction, cerebrovascular thrombosis, and venous thromboembolism. The primary approach in the treatment is both prevention and removal of blood clot. The most effective way to remove blood clot is through the use of thrombin inhibitor or plasminogen activator, which prevent formation of the blood clot or degrade the blood clot, respectively. Indeed, most of drugs developed for CVD treatment are based on either of those approaches. Use of enzymes of therapeutic relevance in treating diseases is increasingly popular, in particular for cardiovascular disease. Thrombolytic agents (enzyme/protein) are classified into two types based on the working mechanisms. Tissue plasminogen activators such as t-PA and urokinase degrade fibrin through activation of plasminogen into active plasmin which in turn will solubilize fibrin in the blood clot and plasmin-like proteins which directly degrade fibrin [8–10].

Several studies have indicated that microorganisms can produce thrombolytic enzyme, which could reduce thrombus formation or act as tissue plasminogen activator and plasmin-like protein [19, 23]. Recently, microbial fibrinolytic enzymes of food origin receive a lot of attention. This situation opens opportunity to explore traditional fermented foods that have been used to treat diseases related to thrombosis. Numerous potent fibrinolytic microorganisms have been isolated from traditional fermented food, such as Korean* Chung Gook-Jang*, Japanese* natto*, Chinese* Douchi*, and Indonesian* Tempe* [2, 11–14].

We have successfully isolated fibrinolytic microorganisms from local (Indonesian) fermented soy bean* Oncom*. Among the microorganisms, a potent isolate was identified as* Stenotrophomonas* sp. which is unique, because most of food origin fibrinolytic microorganism reported belongs to* Bacillus* sp. The extracellular enzymes can degrade fibrinogen in the serum component completely, suggesting the potential application in the process of blood clot solubilization (unpublished data). The fibrinolytic activity was similar to that demonstrated by lumbrokinase (LK) from earthworms* Lumbricus rubellus* which is known for high fibrinolytic activity [10, 19, 23]. The finding of this bacteria upon screening of microbes with fibrinolytic activity is surprising, since genus* Stenotrophomonas* is not yet well explored, but has been associated with variety of beneficial functions and applications, such as plant growth.

Qualified and affordable thrombolysis drugs are urgently needed to reduce the incidence of CVD without neglecting the safety and efficacy of new drugs candidates. The safety studies are usually initiated by using cell culture experiment before proceeding to experimental animals, to avoid conditions that might be harmful to the animal models. In this experiment, enzymes from the three isolates were tested using HeLa S3 and 3T3 cells. The enzyme from isolate* Stenotrophomonas* sp. appeared as somewhat least toxic compared to other enzymes. Therefore, we used this enzyme for further animal experiment.

In this study, we observed higher t-PA expression in HeLa S3 cells, when incubated with fibrinolytic enzyme from* Stenotrophomonas* sp. The expression of t-PA gene can be regulated by both transcriptional and translational mechanisms [4]. Several studies have indicated that t-PA promoter in HeLa cells identified two keys of regulatory region: the first one is related to cAMP responsive element (CRE), and the other is related to sequence similarity to AP2. The expression of t-PA is also regulated by a variety of effectors including cytokines tumor necrosis factor, interleukin, epidermal growth factor, and retinoid tumor promoters [4, 24]. Inflammation can induce plasminogen activator inhibitor-1 (PAI-1) expression that will rapidly induce thrombosis, by activating the coagulation pathway [25].

Carrageenans are family of linear sulphated polysaccharides extracted from red seaweeds [23]. Carrageenan can be used to establish a mouse thrombolytic model involving blackening of the tail due to local inflammation and cell damage to necrosis [13, 26–28] and induced colonic inflammation with development of inflammatory infiltrates, ulceration, and colitis [29]. Necrosis of the tail is associated with vascular thrombosis [20] when carrageenan is administered systemically, due to its agglutinating activity against blood cells [30]. Infarcted tails of rats after carrageenan injection for induction of thrombosis were also shown in previous study to evaluate the fibrinolytic activity of nattokinase [31]. In our study, inflammation and blackening tail were found soon after carrageenan injection. These symptoms were much reduced by oral treatment of the fibrinolytic enzymes from* Stenotrophomona*s sp. and lumbrokinase. Similar result was observed by oral treatment of nattokinase, a fibrinolytic enzyme from* Bacillus natto* isolated from the Japanese traditional fermented food Natto [2, 11]. Apparently, oral treatment of fibrinolytic enzyme for a suitable period of time could be effective in reducing thrombus in the animal organ. The ability to maintain fibrinolytic activity at the animal stomach was observed with another fibrin degrading enzyme, namely, lumbrokinase from the earthworm [10, 19, 32]. The enzyme appeared to be effectively absorbed across the rat intestinal tract [32]. Electron microscopic analysis in our study confirmed the ability of* Stenotrophomonas* enzyme given orally to reach and actively reduce thrombus formed in the rats tail. Safety of the experimental animals following enzyme treatment in our study is reflected by the normal blood parameters observed at the end of experiment.

## 5. Conclusion

In conclusion, we confirmed the safety of enzyme (applied as crude or semipurified form) from* Stenotrophomonas* sp. isolated from Indonesian fermented food* Oncom* in cell culture and experimental rats. The effects of crude and semipurified enzyme on thrombus degrading activity were also similar. The potent thrombolytic activity of the enzymes was shown as significant reduction of kappa carrageenan induced thrombus formation in our experimental rat and also shown through scanning electron microscopy examination.

## Figures and Tables

**Table 1:** Specific activity of fibrinolytic enzyme produced by *Stenotrophomonas *sp.

Sample	Protein (mg/mL)	Specific activity (FU)	Purification factor (FU/mg protein)
Crude enzyme	6.378	0.897	1.000
Am. sulphate (semipurified enzyme)	9.321	1.340	1.024

**Table 2:** Blood parameter.

Blood parameters	Group				
	Normal control group	Negative control group	Positive control group	Crude enzyme of *Stenotrophomonas* sp.	Semipurified enzyme of *Stenotrophomonas* sp.
RBC	6.72 ± 0.28	6.77 ± 0.35	6.59 ± 0.22	6.79 ± 0.32	7.44 ± 0.45^a^
WBC	4.21 ± 0.49	5.21 ± 2.08	6.07 ± 1.71	7.34 ± 1.36	8.18 ± 2.41^a^
PLT	708.10 ± 35.8	942.00 ± 97.89^a^	913.60 ± 97.64^a^	875.10 ± 79.66^a^	851.90 ± 70.65
HGB	13.67 ± 0.41	13.21 ± 0.90	13.75 ± 0.56	12.38 ± 0.66	14.84 ± 0.51
HCT	36.60 ± 1.00	35.92 ± 2.23^e^	36.38 ± 1.11^e^	36.71 ± 1.93	39.54 ± 1.35^bc^
MCV	54.53 ± 1.11	53.03 ± 1.03	55.21 ± 1.65	54.03 ± 0.70	53.19 ± 1.37
MCH	20.36 ± 0.35	19.51 ± 0.51^cd^	20.87 ± 0.71^b^	20.59 ± 0.41^b^	19.96 ± 0.56
MCHC	37.35 ± 0.20^d^	36.75 ± 0.29^cde^	37.80 ± 0.49^b^	38.13 ± 0.43^ab^	37.54 ± 0.38^b^

^a^ *p* < 0.05 in experimental group compared with normal group.

^b^ *p* < 0.05 in experimental group compared with negative control group.

^c^ *p* < 0.05 in experimental group compared with treatment group and positive control group (lumbrokinase).

^d^ *p* < 0.05 in experimental group compared with treatment group, crude enzyme of *Stenotrophomonas *sp.

^e^ *p* < 0.05 in experimental group compared with treatment group, semipurified enzyme of *Stenotrophomonas *sp.

RBC: red blood cell, WBC: white blood cell, PLT: platelet count, HGB: hemoglobin, HCT: hematocrit, MCV: mean corpuscular volume, MCH: mean corpuscular hemoglobin, and MCHC: mean corpuscular hemoglobin concentration.
